# Complete chloroplast genome sequences of four *Allium* species: comparative and phylogenetic analyses

**DOI:** 10.1038/s41598-019-48708-x

**Published:** 2019-08-22

**Authors:** YuMeng Huo, LiMin Gao, BingJiang Liu, YanYan Yang, SuPing Kong, YuQing Sun, YaHui Yang, Xiong Wu

**Affiliations:** 1 Key Laboratory for Biology of Greenhouse Vegetable of Shandong Province, National Center for Vegetable Improvement (Shandong Branch), Vegetable and Flower Research Institute of Shandong Academy of Agricultural Sciences, Jinan, 250100 China; 2 College of Horticulture and Landscape Architecture, Northeast Agricultural University, Harbin, 150030 China; 3 College of Horticulture Science and Engineering, Shandong Agricultural University, Taian, 271018 China

**Keywords:** Molecular evolution, Plant evolution

## Abstract

The genus *Allium* is one of the largest monocotyledonous genera, containing over 850 species, and most of these species are found in temperate climates of the Northern Hemisphere. Furthermore, as a large number of new *Allium* species continue to be identified, phylogenetic classification based on morphological characteristics and a few genetic markers will gradually exhibit extremely low discriminatory power. In this study, we present the use of complete chloroplast genome sequences in genome-scale phylogenetic studies of *Allium*. We sequenced and assembled four *Allium* chloroplast genomes and retrieved five published chloroplast genomes from GenBank. All nine chloroplast genomes were used for genomic comparison and phylogenetic inference. The chloroplast genomes, ranging from 152,387 bp to 154,482 bp in length, exhibited conservation of genomic structure, and gene organization and order. Subsequently, we observed the expansion of IRs from the basal monocot *Acorus americanus* to *Allium*, identified 814 simple sequence repeats, 131 tandem repeats, 154 dispersed repeats and 109 palindromic repeats, and found six highly variable regions. The phylogenetic relationships of the *Allium* species inferred from the chloroplast genomes obtained high support, indicating that chloroplast genome data will be useful for further resolution of the phylogeny of the genus *Allium*.

## Introduction

The genus *Allium* is one of the largest monocotyledonous genera, containing over 850 species^1–4^. Most of these species are found in temperate climates of the Northern Hemisphere and spread widely across the Holarctic region from the dry subtropics to the boreal zone. *Allium* is characterized by herbaceous geophyte perennials with true bulbs, some of which are borne on rhizomes, and a familiar onion or garlic odour and flavour^3^. This genus contains many economically important species, including garlic, leek, onion, shallot, bunching onion, chives and Chinese chives, which are cultivated as vegetables or spices, and species used as herbal crops, such as traditional medicines and ornamental plants^2,5^.

The classification of *Allium* is clear in high taxonomic categories above genus. This genus is first placed in the family Liliaceae and then in the family Alliaceae of the order Asparagales in APG I and APG II^6,7^. The APG classification system has now been revised to APG III and APG IV, so the genus *Allium* becomes a member of the family Amaryllidaceae, subfamily Allioideae, in the new APG classification system^8–10^. However, at the infrageneric level, the classification of *Allium* is very complex, often controversial and remains in progress. A brief history of the infrageneric classification of *Allium* is provided in a number of studies^2,11,12^. With the development of molecular biological methods, many molecular studies on the classification, phylogeny and origin of *Allium* have been performed and many improvements have been made^1,2,11–21^, especially by using the internal transcribed spacer (ITS) region, *rps16* intron and *matK* sequence to understand the evolutionary processes and taxonomic relationships within the genus. The research methods used in those studies are based on morphological characteristics and partial molecular data that are widely used for the classification of new species in this genus^2,8,19,20,22,23^. All of the above mentioned works have been helpful in establishing and assessing the evolutionary lineages in the genus *Allium*. However, due to close morphological similarities among species, a wide variety of habitats, traditional classifications based on homoplasious characteristics and a large number of new species, the precise taxonomy of *Allium* is poorly understood^2^.

The chloroplast (cp), a key organelle for photosynthesis and carbon fixation in green plants, originates from photosynthetic bacteria that interacted with non-photosynthetic hosts via endosymbiosis^24–26^. Moreover, cps have their own genomes, and the genetic information is inherited maternally in most angiosperms^27,28^. Most cp genomes are circular DNA molecules ranging from 120 to 160 kb in length and are highly conserved in terms of gene content and order^29–32^. These genomes have typical quadripartite structures, in which two identical inverted repeat (IR) segments are separated by either a large or a small single-copy region (LSC and SSC, respectively)^33^.

Due to its highly conserved genome structure and gene content, moderate evolutionary rate, uniparental inheritance and nearly collinear gene order in most land plants, the cp genome has been used for the generation of genetic markers for phylogenetic classification^34–37^, divergence dating^21,38^, and DNA barcoding for molecular identification^39,40^. With the rapid development of next-generation sequencing, it is now convenient and relatively inexpensive to obtain cp genome sequences, allowing whole-plastome analysis to obtain large amounts of valuable information^41^ and allowing further extension of phylogenetic analyses based on one or a few loci to whole-genome-based phylogenomic analyses.

Sequencing of the complete cp DNA genome in *Allium* began in 2013^42^, and to date, five *Allium* species namely, *A*. *prattii*, *A*. *obliquum*, *A*. *victorialis*, *A*. *sativum*, and *A*. *cepa*, have been sequenced (http://www.ncbi.nlm.nih.gov/genome/organelle/). And four species in this genus, namely, *A*. *fistulosum*, *A*. *tuberosum* Rottl. ex Spreng., *A*. *sativum*, and *A*. *cepa* are very important vegetable crops not only in *Allium* but also in all vegetable crops acccording to the FAOSTAT in 2017 (http://www.fao.org/faostat/en/#data/QC). The total harvested area and the total production are approximately 7.16 million hectares and 133.39 million tonnes, respectively. A global review of major vegetable crops ranks these third in area and fourth in production. Moreover, even in the same species, there are different cytoplasmic types, which make them different in chloroplast genome. For example, *Allium* cepa has three cytoplasmic types, which is designated as CMS-S (CMS, cytoplasmic male-sterility), CMS-T and N (Normal). The cp genome is expected to be useful not only in the resolving the deeper branches of the phylogeny, but also in DNA barcoding of molecular identification, screening of genetic resources and breeding.

In the present study, we constructed the whole cp genomes of four *Allium* species, *A*. *fistulosum*, *A*. *tuberosum* Rottl. ex Spreng., *A*. *sativum* and *A*. *cepa*, using next-generation sequencing. The objectives of this study were to 1) establish and characterize the organization of the complete cp genomes from four *Allium* species, 2) conduct comparative genomic studies by combining the whole cp genomes of other *Allium* species from GenBank, 3) explore additional molecular markers based on variations in the whole cp genomes, 4) assess the taxonomic positions of *Allium* species based on the complete cp genomes, 5) serve as a reference for future genome-scale phylogenetic studies of *Allium*.

## Results

### Genome sequencing and assembly for four *Allium* species

Four *Allium* species were sequenced, and 8,255,274–13,393,542 paired-end clean reads were obtained. Three complete cp genomes (*A*. *fistulosum*, *A*. *sativum* and *A*. *cepa*) were directly assembled by NOVOPlasty 2.6.2. *A*. *tuberosum* Rottl. ex Spreng. was not circularized by NOVOPlasty. We assembled this genome using SPAdes 3.11.1 and visualized it with Bandage 0.8.1. According to the “Depth range” (≥500), five merged nodes were selected and used to align with the reference NC_024813.1 in Mummer 3.23. The nodes for which the order had been determined were linked to two super-contigs based on their overlap (Supplementary Fig. S1). Two pairs of primers (p1, p2 and p3, p4) were designed according to the two gaps and their sites information provided in Supplementary Fig. S1. Then, PCR amplification and Sanger sequencing were conducted to fill these gaps. Last, the complete cp genomic sequences were assembled by SPAdes 3.11.1 with the options of “–trusted-contigs” including five node and two gap sequences. Alternatively, the sequences from the five nodes and two gaps could be linked manually according to the alignment graph (Supplementary Fig. S1). As a result, four complete cp genomes had been assembled by using the data from two sequencing platforms (HiSeq 4000 and 2500) and two read lengths (150 and 100 bp) (Supplementary Table S1). Finally, the four complete genomes were evaluated by Qualimap v.2.2.1 using the corresponding paired-end reads. The most obvious difference was that the cp DNA extraction methods, including HSLp and SucDNase, produced higher rates of mapping and mean coverage than the total DNA extraction method (Supplementary Table S2). Additionally, HSLp (high-salt low-pH) method had the highest rate of mapping (74.05% and 92.36%), and it was more effective than SucDNase in isolating cp DNA from other DNA (nuclear DNA and mitochondrial DNA) (Supplementary Table S2). Although its mapping rate was the lowest (3.99), the extraction method of total DNA (*A*. *cepa*) also obtained sufficient sequencing depth (334.02X) and a better assembly result because of a large number of reads and a very small cp genome (Supplementary Table S2). The four new complete cp genome sequences were deposited in GenBank (accession numbers: MK335927, MK335929, MK335928 and MK335926).

### Organization and gene content of nine *Allium* species

**Figure 1:**
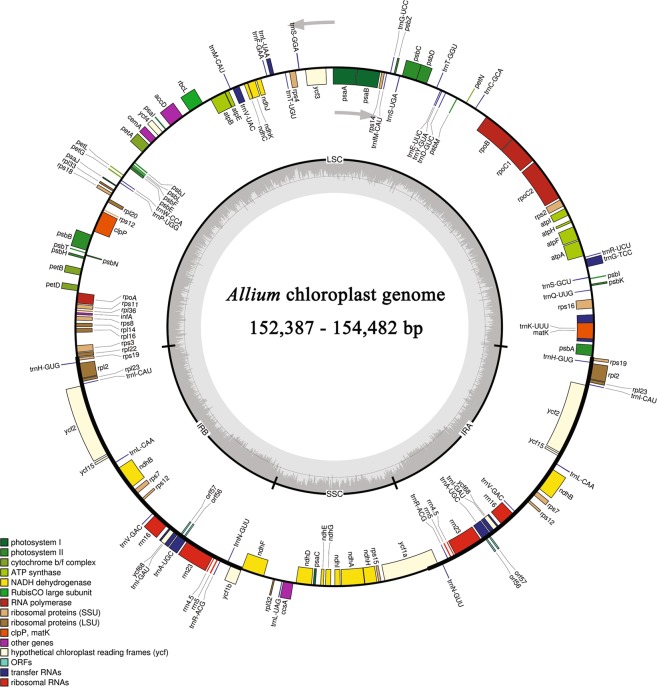
Gene map of the nine *Allium* chloroplast genomes. The genes inside and outside the circle are transcribed in the clockwise and counter-clockwise directions, respectively. Genes belonging to different functional groups are colour coded. The thick lines indicate the extent of the inverted repeats (IRa and IRb) that separate the genomes into large single-copy (LSC) and small single-copy (SSC) regions. Grey bars on the inside of circle indicate GC content, with the line representing 50%.

The nine complete cp genome sequences, which consisted of the four *Allium* species sequenced in this study and five accessions from GenBank, were combined for comprehensive analysis. The genomes ranged in size from 152,387 bp (*A*. *obliquum*) to 154,482 (*A*. *prattii*) (Fig. 1). All of these genomes presented typically quadripartite structures, with two IRs (26,370–26,564) separated by the LSC (81,588–83,392) and SSC (17,853–18,066) regions (Table 1). *Allium* cp genomes showed similar gene content and order, containing 140–141 genes consisting of 88–89 protein-coding genes, 37–38 tRNA genes, 5–10 pseudogene and 8 rRNA genes located in the IR regions (Fig. 1; Table 1). Main components and their proportions were high conserved in eight *Allium* cp genomes except for *A*. *prattii* (Supplementary Tables S3 and S4). However, the numbers and components of pseudogene differed substantially (Table 1; Supplementary Table S5), including those of *atpB*, *ψinfA*, *rps16*, *rps2*, *rbcL*, *trnL-UAA* and *ycf2*. The genes of *atpB*, *rbcL*, *trnL-UAA* and *ycf2* were pseudogenes only in *A*. *prattii*. The pseudogene *infA* was absent in *A*. *tuberosum* Rottl. ex Spreng.. The *rps16* gene was a pseudogene in *A*. *obliquum* and *A*. *sativum* but a protein-coding gene in the other seven cp genomes. The *rps2* gene was a pseudogene in seven accessions (*A*. *fistulosum*, *A*. *tuberosum* Rottl. ex Spreng., *A*. *sativum*, *A*. *cepa* N, *A*. *cepa* CMS-T, *A*. *cepa* CMS-S, and *A*. *obliquum*) but was a protein-coding gene in *A*. *prattii* and *A*. *victorialis*. One tRNA (*trnL-UAA*) was converted to a pseudogene because of the lack of a 5′ end in only *A*. *prattii*.

**Table 1:** Summary of complete cp genomes of *Allium* species.

Species	Genome size	LSC	IR	SSC	Number of genes	Pseudogene	Protein coding gene	tRNA	rRNA	Accession number in Genbank
*A*. *fistulosum*	153,162	82,235	26,510	17,907	141	6	89	38	8	MK335927
*A*. *tuberosum* Rottl. ex Spreng.	154,056	83,068	26,515	17,958	140	5	89	38	8	MK335929
*A*. *sativum*	153,189	82,012	26,564	18,049	141	7	88	38	8	MK335928
*A*. *cepa* N	153,586	82,719	26,468	17,931	141	6	89	38	8	MK335926
*A*. *cepa* CMS-T	153,568	82,702	26,468	17,930	141	6	89	38	8	KM088015
*A*. *cepa* CMS-S	153,440	82,543	26,485	17,927	141	6	89	38	8	KM088014
*A*. *obliquum*	152,387	81,588	26,370	18,059	141	7	88	38	8	NC_037199
*A*. *prattii*	154,482	83,392	26,513	18,066	141	10	86	37	8	NC_037432
*A*. *victorialis*	154,074	83,165	26,528	17,853	141	5	90	38	8	NC_037240

The overall GC content of different regions or components, including complete cp genome, LSC, IR, SSC, coding sequences (CDSs), tRNA, rRNA and pseudogene, was determined based on their annotation. Except for the pseudogene category, the GC content of nine complete cp genomes was very similar in each category (Supplementary Table S6). However, the GC content in eight regions or components of each genome exhibited distinct differences (Supplementary Table S6). The highest was observed in rRNA and the lowest in SSC. The order of the GC content was as follows: rRNA (>55%), tRNA (>52%), IR (>42%), CDSs (>37%), complete genome (>36%), LSC (>34%), SSC (>29%) (Supplementary Table S6). In the pseudogene category, there were relatively large differences among nine *Allium* taxa due to the components and numbers of pseudogenes (*ψinfA*, *ψrps16*, *ψatpB*, *ψrps2*, *ψrbcL*, *ψtrnL-UAA*, *ψycf2*) (Supplementary Tables S5 and S7). *A*. *victorialis* exhibited the highest GC content of 41.04%; *A*. *sativum* the lowest GC content of 35.90%; and three *A*. *cepa* types (N, CMS-T and CMS-S) and *A*. *fistulosum* showed similar GC levels (~39%) (Supplementary Table S6).

### IR/SC boundary

**Figure 2:**
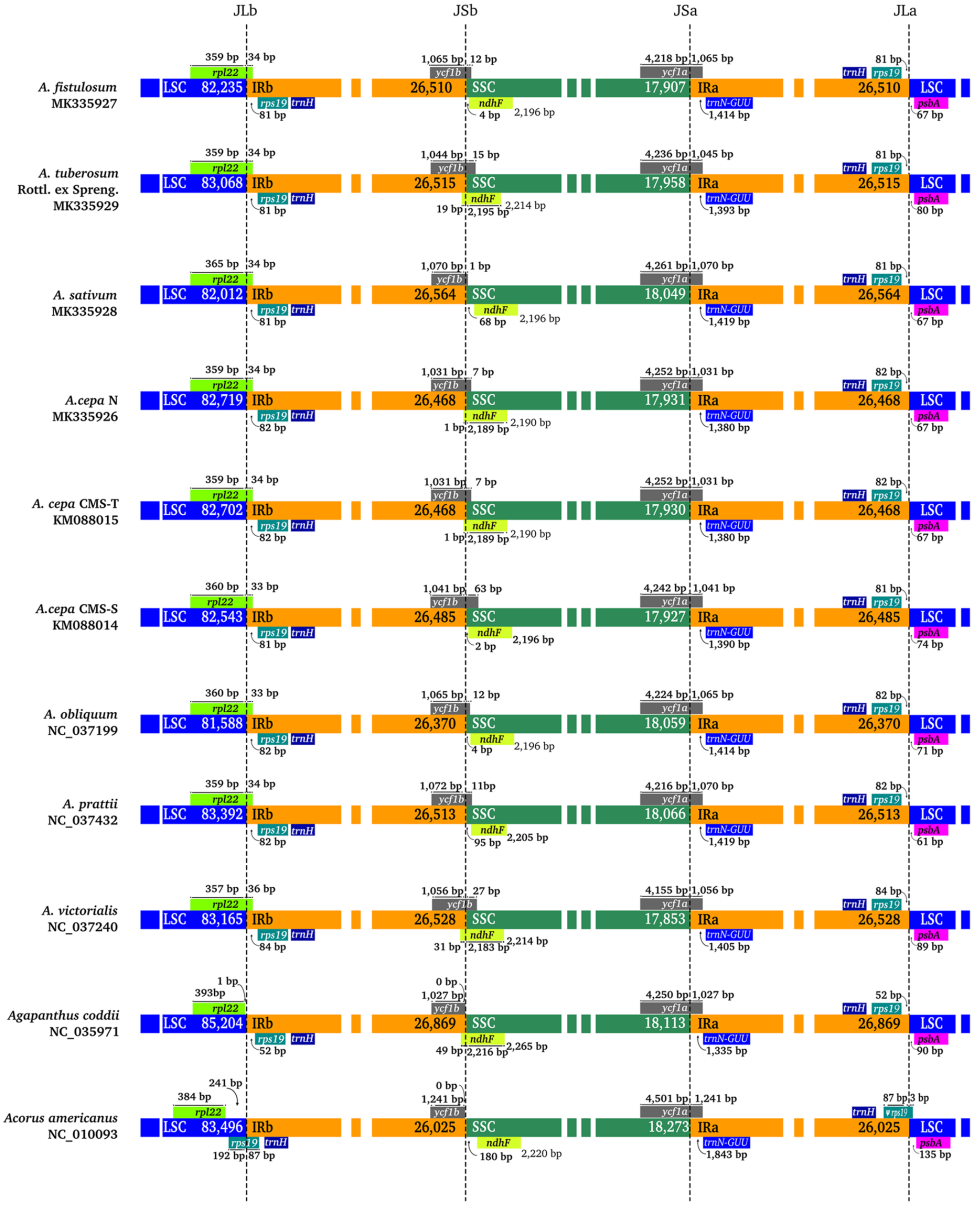
Comparison of the LSC, IR, and SSC boundary regions among the 11 chloroplast genomes. JLa, junction between LSC and IRa; JLb, junction between LSC and IRb; JSa, junction between SSC and IRa; JSb, junction between SSC and IRb. The numbers above the gene features indicate the distances from the end of the gene to the boundary sites. These features are not to scale.

The IR/SC boundary regions of the 11 complete cp genomes were compared, and the IR/SC junctions showed substantial differences (Fig. 2). From basal monocots of *Acorus americanus* to *Agapanthus* or *Allium*, the expansion of the IRs to *rps19* or *rpl22*, which was described using PCR sequences by Wang^43^, was also observed at the IR/ LSC junctions. In *Acorus americanus*, *rps19* flanked the junction between LSC and IRb (JLb), while a partial sequence of *rps19* was present in the IR regions and another located in the LSC region. However, two IRs all contained a complete *trnH-rps19* cluster with a length of 81–84 bp away from the IR/LSC boundary in *Allium* and 52 bp in *Agapanthus coddii*. Then, JLb expanded into the 5′ portion of the *rpl22* gene with a length of 33–36 bp in *Allium*. The junctions of IR/SSC were located in the gene *ycf1*(*a* or *b*) or between *ycf1b* and *ndhF*. The 3′ end of the gene *ycf1b* and *ndhF* exhibited substantial differences for expansion or contraction of IRs. Overlaps of *orf* (*ycf1a* and *ndhF)* were observed in *A*. *fistulosum*, *A*. *tuberosum* Rottl. ex Spreng., *A*. *cepa* (N, CMS-T and CMS-S), *A*. *obliquum*, *A*. *victorialis* and *Agapanthus coddii*. The length from *ndhF* to the junction between SSC and IRb (JSb) exhibited a distinct difference (from 180 to −31), so the boundary characteristics of IR/SSC were more complex than those of IR/LSC. Overall, the IR/SC boundary regions in the nine *Allium* species showed similar characteristics, with only slight differences in the length flanking or away from the boundary in the organization genes, namely, *rpl22*, *rps19*, *ycf1b*, *ndhF*, *ycf1a* and *psbA*.

### Repeat sequence analysis

**Figure 3:**
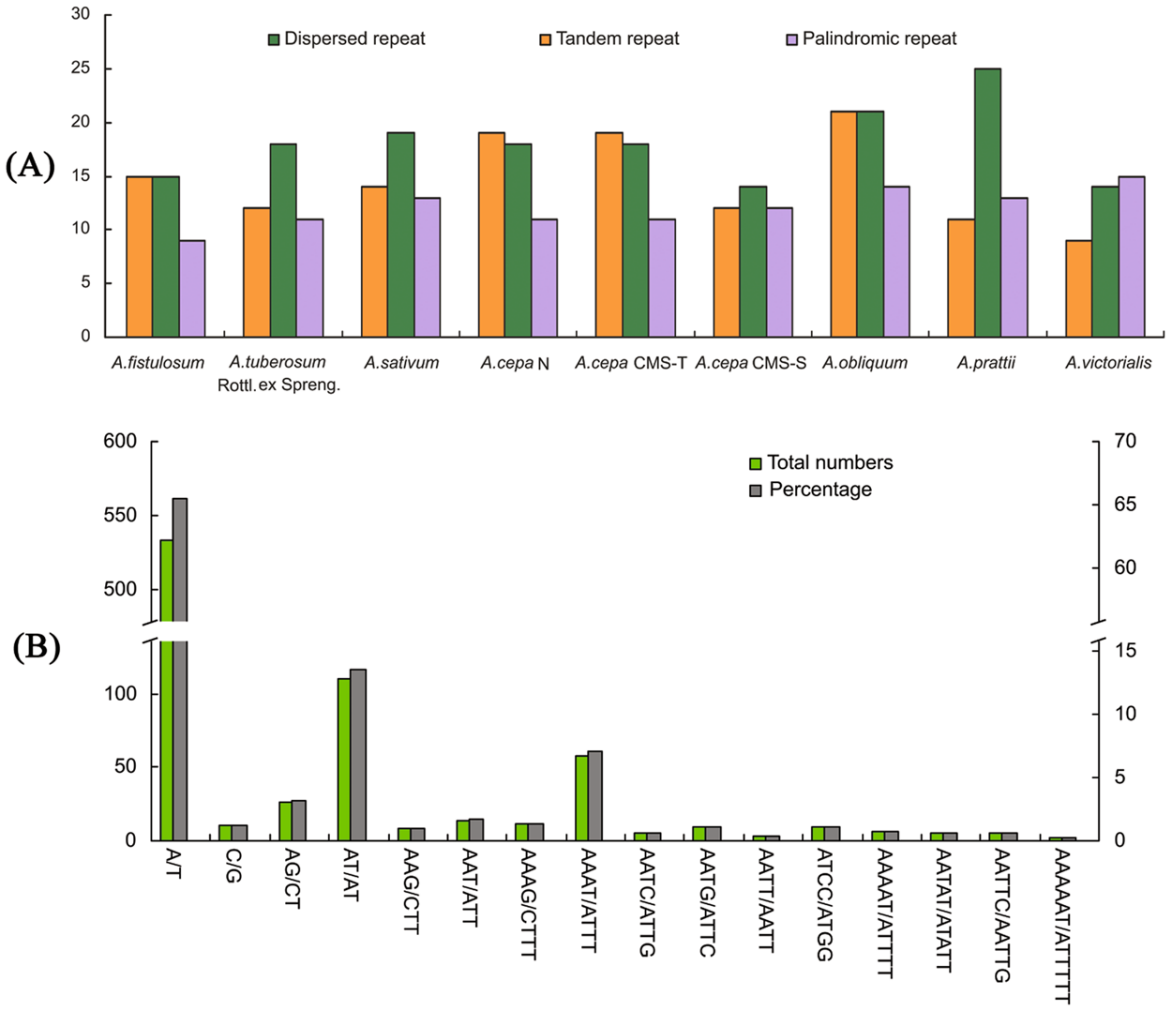
Numbers of the three repeat types in the nine *Allium* cp genomes (**A**) and types and numbers of SSRs (**B**).

The numbers and distributions of three repeat types (tandem, dispersed and palindromic repeats) in the nine *Allium* cp genomes were similar and conserved (Fig. 3A; Supplementary Table S8). There were 394 repeats, including 131 tandem repeats, 154 dispersed repeats and 109 palindromic repeats (Supplementary Table S8). These repeats were distributed in 657 sites containing 131 tandem repeat sites and 526 dispersed and palindromic repeat sites (one site was counted in one tandem repeat and two sites in one dispersed or palindromic repeat) (Supplementary Datasets 1 and 2). The lengths of the repeat units ranged from 11 to 91 bp. Based on the quadripartite structure of the cp genome, LSC regions had the most repeat sites (411, 62.56%), followed by IR (198, 30.14%), SSC (42, 6.39%) and the overhanging junction (6, 0.91%, 1 SSC/IRa and 5 IRb/SSC) (Supplementary Fig. S2A). According to the classification of gene structure, CDS, IGS (intergenic spacer) and intron, a majority of the repeat sites were in IGS regions (451, 68.65%), in which the *ycf2*~*trnI* contained the most numbers of repeat sites (2 × 27, 2 × 4.11%), and a minority were in introns (36, 5.48%) (Supplementary Fig. S2B). Only a few types of gene (e.g., *psaA*, *psaB*, *rpoC2*, *trnF*, *ycf1a*, *ycf1b*, *ycf1b-ndhF*, *ycf2*) possessed repeat elements, and the gene *ycf2* contained the highest number of repeat sites (120, 18.26%). *A*. *obliquum*, with 55 repeats, had the maximum number of repeats, and *A*. *victorialis* and *A*. *cepa* CMS-S, with 37 repeats, had the lowest number of repeats (Supplementary Tables S8).

We detected 814 simple sequence repeats (SSRs) in the nine cp genomes using the Perl script MISA. The numbers of SSRs differ among the nine *Allium* genomes and vary from 73 in *A*. *tuberosum* Rottl. ex Spreng. to 96 in *A*. *cepa* N and T, as shown in Supplementary Table S9. The most abundant SSR motifs were mononucleotide repeats, which accounted for approximately 66.71% of the total SSRs, followed by dinucleotide (16.71%) and tetranucleotide (11.67%) repeats. We also found that there were more tetranucleotide repeats than trinucleotide and pentanucleotide repeats. Hexanucleotide repeats were very rare across these cp genomes, appearing only once in *A*. *tuberosum* Rottl. ex Spreng. and *A*. *cepa* CMS-S. Almost all mononucleotide repeats were composed of A/T (98.16%), with only 1.84% composed of C/G. AT/AT repeats constitute was 80.88% of dinucleotide repeats, while AG/CT repeats constitute only 19.12% (Fig. 3B).

### Sequence divergence analysis

**Figure 4:**
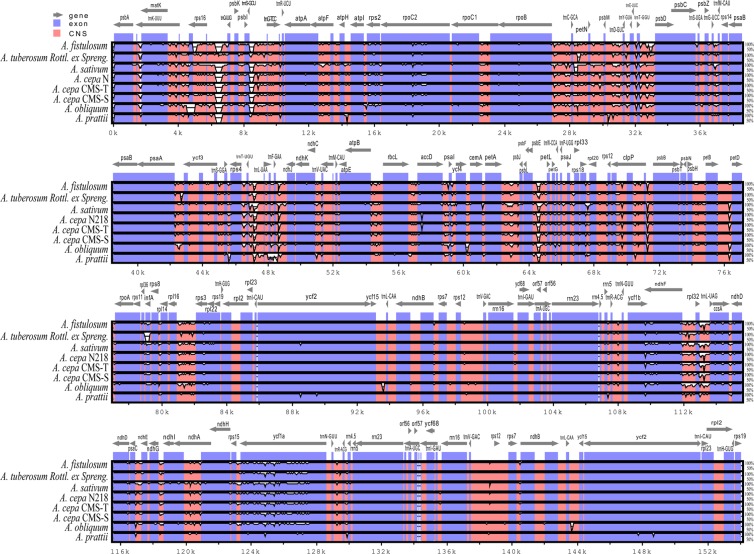
Sequence identity plot for the nine *Allium* chloroplast genomes with *A*. *victorialis* as a reference, as visualized by mVISTA. Grey arrows above the alignment indicate the orientations of the genes. Blue bars represent exons, and pink bars represent non-coding sequences (CNSs). A cut-off of 50% identity was used for the plots. The Y-axis represents the percent identity within 50–100%.

With *A*. *victorialis* as a reference, alignments of the nine complete cp genomes were performed using mVISTA. The results revealed high sequence conservation (97.14–98.22%) across the nine *Allium* cp genomes, especially in gene regions (99.23–99.46%) (Fig. 4; Supplementary Table S10). There were only slight differences in CNGs (conserved non-gene sequences) (93.88–96.62%) (Supplementary Table S10).

**Figure 5:**
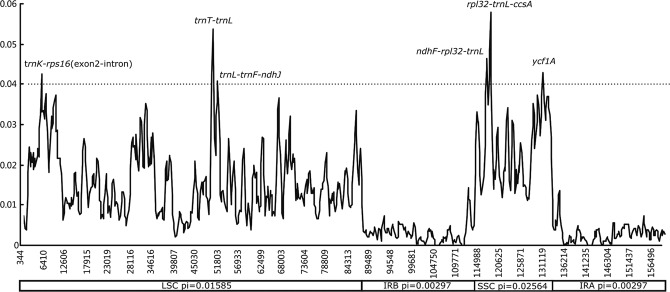
Sliding window analysis of the nine *Allium* chloroplast genome sequences (window length: 600 bp; step size: 200 bp). The Y-axis represents the nucleotide diversity of each window, while the X-axis represents the position of the midpoint.

The nucleotide diversity in the complete cp genome, LSC, IR, and SSC was compared among the nine *Allium* cp genomes. Based on analysis by DnaSP version 6.1 software, polymorphic sites, parsimony-informative sites, and nucleotide diversity were determined. In the complete cp genomes, 5,552 polymorphic sites (3.49%) and 2,502 parsimony-informative sites (1.57%) were observed, and the nucleotide diversity was 0.01244 (Table 2). SSC regions exhibited higher divergence (0.02564) than LSC (0.01585) and IR (0.00297) regions (Table 2). To further calculate the sequence divergence level in the local regions of cp genomes, the nucleotide diversity (pi) value within a 600-bp window was calculated with 200-bp steps. These values varied from 0 to 0.05787. Then, six highly divergence regions (or hotspot regions) (Table 3), namely, *trnK*-*rps16* (exon2-intron), *trnT*-*trnL*, *trnL*-*trnF*-*ndhJ*, *ndhF*-*rpl32 -trnL*, *rpl32-trnL*-*ccsA*, and *ycf1a*, were identified with a cut-off of 0.04. These hotspots were all located in SC (LSC and SSC) regions (Fig. 5), and only *ycf1a* was in the coding region. The *rpl32-trnL* region exhibited the highest variability (0.05787).

**Table 2:** Variable site analyses in *Allium* cp genomes. ccpg, complete chloroplast genome.

Region	Total number of sites	Polymorphic sites	Parsimony informative sites	Nucleotide diversity
ccpg	159,150	5,552	2,502	0.01244
LSC	87,217	3,732	1,679	0.01585
IR	26,652	238	114	0.00297
SSC	18,703	1,338	590	0.02564
SC	105,861	5,076	2,270	0.01764

**Table 3:** Six regions of highly variable sequence of *Allium*. e2, exon2. i, intron.

High variable region	Length	Polymorphic sites	Parsimony informative sites	Nucleotide diversity
*trnK*-*rps16*(e2-i)	1,379	71	37	0.04245
*trnT*-*trnL*	1,425	114	36	0.04354
*trnL*-*trnF*-*ndhJ*	1,368	91	18	0.04097
*ndhF*-*rpl32-trnL*	1,614	153	67	0.04319
*rpl32*-*trnL*-*ccsA*	731	65	35	0.04088
*ycf1a*	624	77	36	0.04296
Combine	7,141	571	229	0.04253

The p-distance and number of nucleotide substitutions were used to estimate divergence among the nine *Allium* cp genomes. The p-distance ranged from 0.00006 to 0.02306 with an overall average of 0.01244, and the number of nucleotide substitutions was found to range from 9 to 3,411 (Supplementary Table S11). *A*. *prattii* and *A*. *sativum* exhibited the greatest sequence divergence (0.02306). *A*. *cepa* N exhibited only nine nucleotide substitutions compared to *A*. *cepa* CMS-T but 316 nucleotide substitutions compared to *A*. *cepa* CMS-S. These results further indicated that the onion species with N and CMS-T cytoplasm were more closely related to each other than to that with a CMS-S cytoplasm.

### Phylogenetic analysis

**Figure 6:**
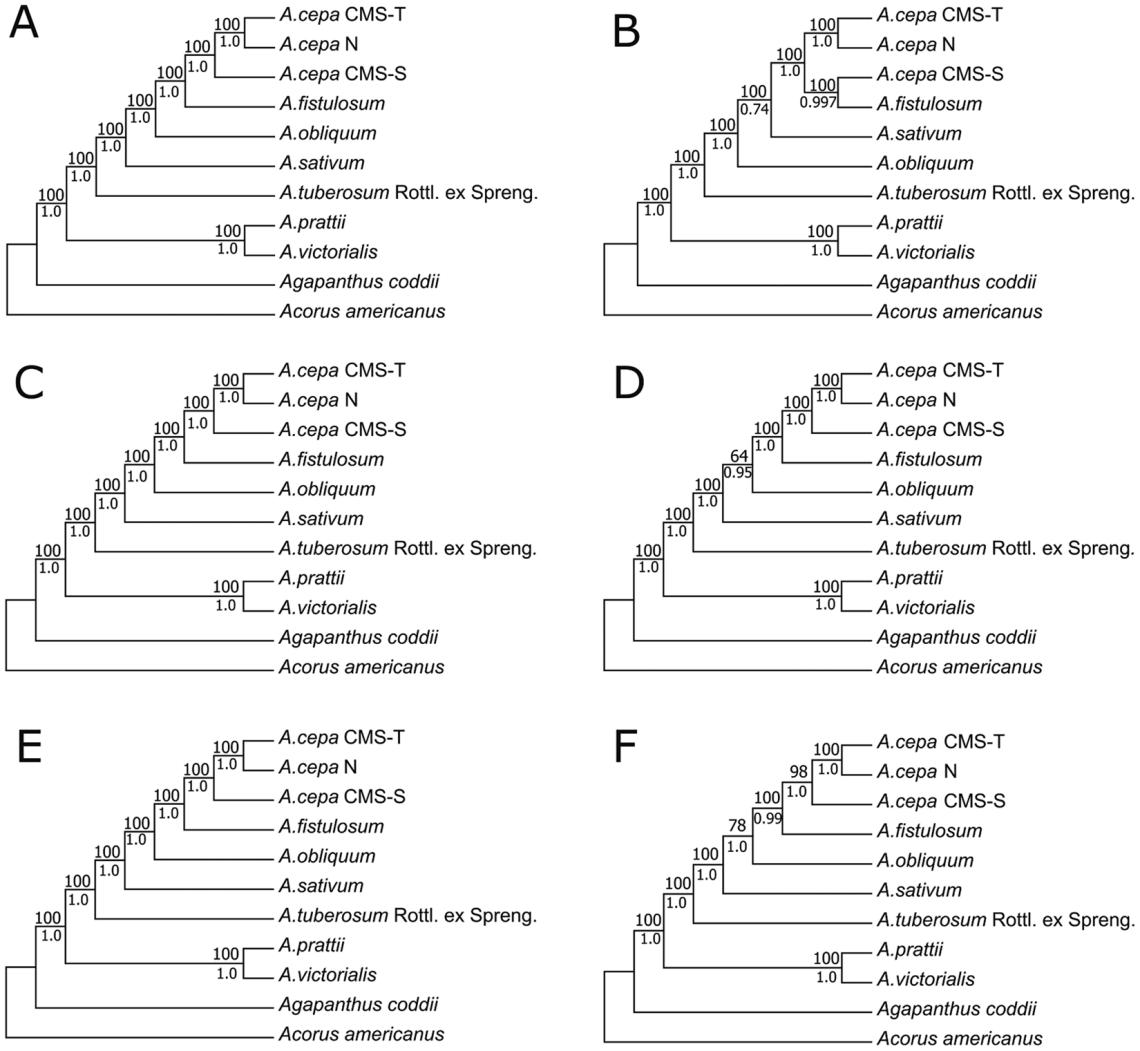
Phylogenetic relationships of the nine *Allium* species inferred by maximum likelihood (ML) methods and Bayesian inference (BI) analyses of different datasets. (**A**) Complete chloroplast genome. (**B**) IR region. (**C**) LSC region. (**D**) SSC region. (**E**) SC region. (**F**) Six divergence hotspots. The numbers associated with each node are bootstrap support values (above the node) for ML and posterior probability values (under the node) for BI in (**A**–**F**).

In this study, six datasets (complete chloroplast genome, IR, LSC, SSC, SC and the combined variable regions) from 11 cp genomes sequences were created on the basis of their annotation, and the number of sites used to construct phylogenetic trees ranged from 3,724 to 137,185 (Supplementary Table S12). According to the identification results obtained by jModeltest v2.1.10, the best-fit models for each dataset based on the Akaike information criterion (AIC) are listed in Supplementary Table S12. The maximum likelihood (ML) and Bayesian inference (BI) models were selected based on the above results and the RAxML v8.2.12 manual. The topologies of the phylogenetic trees based on the two methods of analysis (ML and BI) were identical for each dataset. And the datasets generated similar topological structures with a very high support, except for the IR dataset (Fig. 6). *Allium* species and *Agapanthus coddii* produced two distinct branches with very high support (100% and 1.00). In the genus *Allium*, nine accessions were divided into two sister clades. The first clade contained two species, namely, *A*. *prattii* and *A*. *victorialis*. The 2nd clade included seven accessions, in which *A*. *cepa* (CMS-T and N) was grouped in a sister branch and then clustered step by step with *A*. *cepa* CMS-S, *A*. *fistulosum*, *A*. *obliquum*, *A*. *sativum* and *A*. *tuberosum* Rottl. ex Spreng.

From the dataset of the divergence hotspots (including only 229 parsimony-informative sites) (Table 3), we also inferred the phylogenetic relationships that were identical to other datasets except for IR dataset, in terms of topological structure (Fig. 6). Moreover, even in the infraspecific classification, three cytoplasmic types of *A*. *cepa* were also clearly identified. The cp genome can be used to resolve the deeper branches within species. However, the present study analysed only a limited number of species. With the rapid improvement of sequencing technologies, the sequencing of complete cp genome will become routine. Therefore, more and more complete sequences of cp genome will be used to further elucidate the phylogenetic relationships of the genus *Allium*.

## Discussion

In this study, four complete cp genome sequences were sequenced and assembled. The genome sizes ranged from 153,162 bp (*A*. *fistulosum*) to 154,056 bp (*A*. *tuberosum* Rottl. ex Spreng.), similar to the genome size for *A*. *cepa* reported by Kohn^42^ and Kim^44^. Subsequently, nine complete *Allium* cp genome sequences, including those of the four *Allium* species sequenced in this study and five obtained from GenBank, were compared. The cp genomes of *Allium* are highly conserved, with identical gene content and order, and genomic structures comprising four parts^33^. The GC levels of the complete cp genomes were very similar, ranging from 36.7 to 37.0% (Supplementary Table S6), which has also been observed in other angiosperm cp genomes^42,44,45^. Additionally, the *rps12* gene is a trans-spliced gene with the 5′ end located in the LSC region and the duplicated 3′ end in the IR region, as has been identified previously in other reports^42^. However, the numbers and components of pseudogene were substantially different, especially the loss of sequence *infA* in *A*. *tuberosum* Rottl. ex Spreng.. In addition, surprisingly, the genes *atpB*, *rbcL*, *trnL-UAA* and *ycf2* were present as pseudogene in only *A*. *prattii* but as protein-coding genes in the other eight cp genomes (Supplementary Table S5). This transformation might be caused by sequence contamination originating from the mitochondrial genome^46,47^ or by annotation error, as previously discussed for Fagaceae^48^. Due to the components and numbers of pseudogene, the GC levels and lengths of the pseudogene also varied by species from 35.9% to 41.04% and 1,043 to 17,782, respectively (Supplementary Table S7).

The change in position of the IR/SC junction may have been caused by contraction or expansion of the IR region, which is a common evolutionary phenomenon^34,43,49–51^ and may cause variations in the lengths of angiosperm plastid genomes^49^. In the nine *Allium* species, the IR/SC boundary regions showed similar characteristics, with only slight differences observed in the length flanking or away from organization genes, namely, *rpl22*, *rps19*, *ycf1b*, *ndhF*, *ycf1a* and *psbA* (Fig. 2). Expansion of IR regions was also found from basal monocots of *Acorus americanus* to *Agapanthus* or *Allium*^43^. The complete *trnH-rps19* cluster is present in IR regions, in which this type of IR/LSC junction is consistent with TYPE III, as reported by Wang^43^. We also found that *A*. *cepa* N and CMS-T exhibited the same features in four junctions, but *A*. *cepa* CMS-S exhibited slight differences compared with *A*. *cepa* N and CMS-T. For example, the length of extension of *rpl22* into IRb was 34 bp in N and CMS-T and 33 bp in CMS-S. These differences in length were also exhibited by other organization genes, such as *rps19*, *ycf1b*, *ycf1a*, *ndhF* and *psbA*. These results maybe hint the difference in origin and evolution by previous reports^44,52–56^.

Large, complex repeat sequences may play important roles in the rearrangement of plastid genomes and sequence divergence^57,58^. We found that the repeat sites in the nine *Allium* species were similar and conserved, usually located in IGS regions (451, 68.65%) (Supplementary Fig. S2). Surprisingly, the *ycf2* gene contained the most repeat sites (120, 18.26%), and the *ycf1* gene contained only seven sites (1.07%) (Figs S2 and S3). Moreover, we identified 81 long repeats of more than 40 bp, accounting for approximately 20.56% of the total 394 repeats. This rate is similar to previous reports on other plant lineages^34,59,60^. SSRs are thought to be the results of slipped strand mispairing during DNA replication, which are frequently observed in cp genomes and have been shown to have substantial application potential in population genetics and breeding programmes^61–63^. In this study, 814 SSRs were identified, with the most abundant mononucleotide repeats, accounting for 66.71% of the total SSRs, followed by dinucleotide, tetranucleotide, trinucleotide, pentanucleotide, and hexanucleotide repeats. Almost all mononucleotide repeats were composed of A/T (98.16%), with only 1.84% composed of C/G. Among dinucleotide repeats, AT/AT accounted for 80.88%, while AG/CT for only 19.12%. Our results are comparable to previously reported findings that SSRs in cp genomes are composed of polyadenine (poly A) or polythymine (poly T) repeats and rarely contained tandem guanine (G) or cytosine (C) repeats^45^. We also found that tetranucleotide repeats were more abundant than trinucleotide and pentanucleotide repeats, which is consistent with a report on *Quercus* species^64^. Hexanucleotide repeats were very rare across the nine *Allium* cp genomes, similar to the results in *Lilium*^45^. These new SSR resources will potentially be useful for population studies on the *Allium* genus, especially in combination with other informative nuclear genome SSRs.

The alignments of the nine *Allium* complete cp genomes revealed a high degree of synteny (Fig. 4). SC regions were more divergent than two IR regions, and the non-coding regions exhibited greater divergence than the coding regions. Similar results are reported previously for many cp genomes^31,39,45,64^. Differences in *accD* in S/N-cytoplasmic onions, which were detected in a previous report^42^, were also observed (Fig. 4). The nucleotide substitution rate is a central feature of molecular evolution^65^. All pair-wise sequence comparisons in our study showed that the number of nucleotide substitutions differed greatly, ranging from 9 to 3,411 (Supplementary Table S11). This result suggests that DNA sequences evolve at different rates in different species which had also been observed in other taxa^66^.

Six highly divergence regions (Table 3), namely, *trnK*-*rps16* (exon2-intron), *trnT*-*trnL*, *trnL*-*trnF*-*ndhJ*, *ndhF*-*rpl32-trnL*, *rpl32-trnL*-*ccsA*, and *ycf1a*, were identified with a cut-off of 0.04. These regions exhibited far greater nucleotide diversity than *matK* and *rps16* previously reported in evolutionary issues and taxonomic relationships^1,2,11,12,14–21^. Based on these results, we believe that these regions, which exhibit relatively high sequence deviation, might be regarded as potential molecular markers and are useful resources for interspecies and infraspecific phylogenetic analysis of *Allium* species.

The phylogenetic trees based on different datasets produced similar topological structures, except for the IR dataset, possibly because this region was highly conserved and provided fewer informative sites than the SC regions (Table 2). First, *Allium* species and *Agapanthus coddii* produced two distinct branches with very high support (100% and 1.00). Then, in the genus *Allium*, nine taxa were divided into two clades. The first clade included two species (*A*. *prattii* and *A*. *victorialis*) belonging to the second evolutionary line described in previous reports^2,11,22^. The other species, including *A*. *tuberosum* Rottl. ex Spreng., *A*. *sativum*, *A*. *obliquum*, and *A*. *cepa* (CMS-T, CMS-S and N), grouped into clade two, belonging to the third evolutionary line^2,11,22^. In *A*. *cepa*, CMS-T and N grouped into a sister branch and then clustered with CMS-S. These results indicate that CMS-T and N share a close relationship and that CMS-S does not originate from *A*. *cepa* N and T, which is consistent with previous reports that the S cytoplasm had two origins^67^. The S cytoplasm may be an alien cytoplasm transferred from an unknown *Allium* species to the bulb onion through the viviparous interspecific triploid ‘Pran′. Alternatively, the S cytoplasm could be a component of one or more wild populations from which onion was domesticated, and S-cytoplasmic plants could be the seed parents of ‘Pran’^67^.

The results presented here not only robustly support previous reports of three major clades inferred by ITS, *rps16* and *matK* data^1,2,11,12,14–21^ but also show increased bootstrap or posterior probability values, especially at low taxonomic levels (such as infraspecific classification). These results also suggest that cp genome data can effectively resolve the phylogenetic relationships of the genus *Allium*. Although the *matK*, *rps16*, and ITS genes have been widely used to investigate taxonomy in *Allium*, these markers will exhibit increasingly extremely low discriminatory power because this genus contains more than a lot of species and especially a large number of new *Allium* species are being identified step by step^11,21^. Fortunately, for the purposes of this study, the cp genomes of *Allium* species have highly divergent regions with far greater nucleotide diversity than *matK* and *rps16*. Moreover, using the dataset of highly divergent regions, we also inferred the deeper phylogenetic relationships that were identical with other datasets, except for the IR dataset. This finding also suggests that these regions are useful resources for the phylogenetic analysis of *Allium* species, not only in interspecies but also infraspecific classification. Although the present study analyzed a limited number of species, our data will serve as a reference for future genome-scale phylogenetic studies of *Allium*. With the rapid improvement in sequencing technologies, sequencing of complete cp genome will become routine. Therefore, an increasing number of cp genome sequences will be used to further elucidate the phylogenetic relationships of the genus *Allium*.

## Methods

### Plant material and DNA extraction

Fresh leaves of four *Allium* species were harvested from the Vegetable and Flower Research Institute of Shandong Academy of Agricultural Sciences. DNA samples were isolated by three methods (Supplementary Table S1): (1) Total genomic DNA for *A*. *cepa* N (N218) was isolated using the Plant Genome Extraction Kit (PGEK) (Tiangen Biotech, Beijing, China); (2) cpDNA for *A*. *tuberosum* Rottl. ex Spreng. was isolated by the sucrose-DNase (SucDNase) method^68^; (3) cpDNA for *A*. *fistulosum* and *A*. *sativum* was isolated by the high-salt low-pH (HSLp) method^69^. DNA concentration and quality were measured using a NanoPhotometer P330 (Implen GmbH, Munich, Germany) and agarose gel electrophoresis.

### Genome sequencing, assembly, and annotation

DNA samples from *A*. *fistulosum*, *A*. *tuberosum* Rottl. ex Spreng. and *A*. *sativum* were sheared to construct a ~350-bp paired-end library in accordance with the Illumina HiSeq 4000 protocol to obtain an average read length of 150 bp (Supplementary Table S1). Another ~350-bp paired-end library for the *A*. *cepa* sample was constructed using the Illumina HiSeq 2500 protocol with an average read length of 100 bp (Supplementary Table S1). Quality control of the raw sequence reads was performed using an ultra-fast FASTQ preprocessor, fastp version 0.15.0^70^, using default parameters, except -q 20 and -n 10. Each species yielded at least 1.2 Gb of clean data (Supplementary Table S2).

First, high-quality reads were assembled by NOVOPlasty 2.6.2^71^ with the default parameters set using the seed sequence *AcrbcL* from the reference sequence NC_024813.1. The orientation was resolved manually based on NC_024813.1. *A*. *tuberosum* Rottl. ex Spreng. was not circularized by NOVOPlasty, and therefore, we assembled this sequence using SPAdes 3.11.1^72^. The file of “fastg” was visualized by the software Bandage 0.8.1^73^, and the alignment of nodes or contigs were conducted by Mummer 3.23^74^. Gaps in the cp genome sequences were filled by PCR amplification and Sanger sequencing based on reference NC_024813.1. PCR was performed in a total volume of 25 μl using the TaKaRa PCR Amplification Kit (TaKaRa Biotechnology, Dalian, China). The PCR mixtures contained 50 ng template DNA, 0.2 μM of each primer, and 12.5 μl PCR Mix. The primer pairs p1 (5′ GAGACTACCAGATCCCCGCTAT 3′) and p2 (5′ CTTTGGAATACTGGAAGGGTCG 3′) were used to amplify gap1, and p3 (5′ ATGTCGAATACTAACTTATCTGTCTGC 3′) and p4 (5′ ATTTCACCATAGCGGCTTACTT 3′) to gap2. The PCR protocol was as follows: initial denaturation at 94 °C for 4 min, followed by 35 cycles of 94 °C for 30 s, 50 °C for 30 s, 72 °C for 1 min, with a final 5 min extension at 72 °C. The amplified products were separated on 1.0% agarose gels and visualized by ethidium bromide staining. Evaluation of the assembly was performed by Qualimap v.2.2.1^75^.

The complete cp genomes were annotated by plann v.1.1.2^76^ using NC_024813.1 as a reference and then checked by DOGMA^77^ (http://dogma.ccbb.utexas.edu/). The positions of start and stop codons, and the boundaries between introns and exons were manually corrected by comparison with the published cp genome of NC_024813.1. The annotated GenBank files were used to draw circular cp genome maps using OrganellarGenome DRAW (https://chlorobox.mpimp-golm.mpg.de/OGDraw.html). The organization and gene content of the nine *Allium* taxa were analysed according to the corresponding annotations. Then, the boundary regions of the LSC, SSC, and IRs were also compared from 11 accessions, including the nine *Allium* cp genomes and those of the closely related species *Agapanthus coddii*, and the basal monocot *Acorus americanus*.

### Repeat element analysis

Tandem repeats were detected using Tandem Repeats Finder (TRF) version 4.09^78^ with advanced parameters. The alignment parameters match, mismatch, and indel were set to 2, 7, and 7, respectively, and the minimum alignment score and maximum period size were set to 80 and 500, respectively. Other parameters were set to default values. The Perl script repfind.pl from Vmatch^79^ was used to find dispersed and palindromic repeats in which the minimal repeat size was 30 bp and the two repeat copies had at least 90% similarity (i.e., a Hamming distance of 3, -h 3). Then, two types of repeats were sorted by Vmatch with the -d (or -p, separately) -l 30 -h 3 -sort ia options. Complete IRa and IRb were excluded from the palindromic repeats. The Perl script MISA^80^ was used to detect SSRs or microsatellites. The minimum numbers of repeats were 10, 5, 4, 3, 3, and 3 for mono-, di-, tri-, tetra-, penta-, and hexanucleotide repeats, respectively.

### Sequence divergence analysis

The complete cp genomes were compared using the mVISTA program^81^ with *A*. *victorialis* as a reference, because of the least numbers of pseudogenes and the second length cp genome. Then, the sequences were first aligned using MAFFT v7.394^82^ and manually adjusted in MEGA v7.0.26^83^. Subsequently, a sliding window analysis was conducted to evaluate the nucleotide variability (Pi) of the cp genome using DnaSP v6.12.01 software^84^. The step size was set to 200 bp, and the window length was set to 600 bp. Variable and parsimony-informative base sites across the complete cp genomes and the LSC, SSC, and IR regions of the nine cp genomes were calculated. The p-distance and number of nucleotide substitutions among *Allium* cp genomes were calculated using MEGA v7.0.26^83^ software.

### Phylogenetic analysis

Phylogenetic analysis was conducted on the basis of 11 accessions, including the four species in the current study, five other *Allium* species (*A*. *obliquum*, *A*. *prattii*, *A*. *victorialis*, *A*. *cepa* CMS-T and *A*. *cepa* CMS-S), and *Agapanthus coddii*, belonging to the genus *Agapanthus*, which is closely related to *Allium*. *Acorus americanus* was used as an out-group. Because molecular evolutionary rates differed among the different cp genome regions, six datasets were created according to the complete cp genome annotation described above, consisting of (A) complete chloroplast genome, (B) IR region, (C) LSC region, (D) SSC region, (E) SC region and (F) the combined variable regions. All sequences were aligned using MAFFT v7.394^82^, and all alignments were manually adjusted in MEGA7.0.26^83^. All gap positions were eliminated by Gblocks v0.91b^85^. All phylogenetic analyses were performed using maximum likelihood (ML) methods and Bayesian inference (BI). The best-fit substitution models were selected by the AIC for ML trees and the Bayesian information criterion (BIC) for BI trees in jModeltest v2.1.10^86,87^. ML analysis was performed using RAxML v8.2.12 with 1,000 rapid bootstrap replicates^88^. BI was implemented with MrBayes v3.2.6^89^. Two independent Markov chain Monte Carlo (MCMC) chains were run, each with three heated and one cold chain for 10 million generations (Ngen = 10,000,000). All trees were sampled every 1,000 generations (Samplefreq = 1,000). Stationarity was considered to be reached when the average standard deviations of the split frequencies remained below 0.01. The first 25% of the trees were discarded as burn-in, and the remaining trees were used to build a majority-rule consensus tree.

### Accession code

The four complete cp genome sequences of *Allium* species (*A*. *fistulosum*, *A*. *tuberosum* Rottl. ex Spreng., *A*. *sativum* and *A*. *cepa*), were deposited in GenBank (accession numbers: MK335927, MK335929, MK335928 and MK335926).

## Supplementary information

Supplementary Table S1-S12 and Figure S1-S3

Dataset 1

Dataset 2
