# Supplementary material for: Complete chloroplast genome sequences of four Allium species: comparative and phylogenetic analyses
Source: Sci Rep. 2019 Aug 22;9:12250. doi: 10.1038/s41598-019-48708-x (PMC6706373; doi:10.1038/s41598-019-48708-x)
Supplement: Supplementary file 1 — Supplementary Table S1-S12 and Figure S1-S3 [file 41598_2019_48708_MOESM1_ESM.pdf]

Complete chloroplast genome sequences of four *Allium* species: comparative and phylogenetic analyses

YuMeng Huo[1], LiMin Gao[1], BingJiang Liu[1], YanYan Yang[1], SuPing Kong[1], YuQing Sun[2], YaHui Yang[3], Xiong Wu[1,*]

[1]Key Laboratory for Biology of Greenhouse Vegetable of Shandong Province, National Center for Vegetable Improvement (Shandong Branch), Vegetable and Flower Research Institute of Shandong Academy of Agricultural Sciences, Jinan, 250100, China

[2]College of Horticulture and Landscape Architecture, Northeast Agricultural University, Harbin, 150030, China

[3]College of Horticulture Science and Engineering, Shandong Agricultural University, Taian, 271018, China

*Corresponding Author: Xiong Wu

Telephone and Fax: +86(0)531-6665-9755

Email address: wutta2014@163.com

Supplementary Tables

Supplementary Table S1. Basic information about the library, DNA extraction and assembly.

Supplementary Table S2. Summary of the sequencing data and assembled evaluation for four *Allium* species.

Supplementary Table S3. List of genes in nine cp genome of *Allium* species.

Supplementary Table S4. Main components and their proportions in nine *Allium* cp genomes.

Supplementary Table S5. List of the difference in genes content from nine *Allium* cp genome.

Supplementary Table S6. GC content (%) of sequence in nine cp genomes of *Allium* species.

Supplementary Table S7. The content, length (bp) and GC (%) of pseudogene in *Allium* species.

Supplementary Table S8. The numbers of tandem repeats, dispersed, and palindromic in nine *Allium* cp genomes.

Supplementary Table S9. Types and numbers of SSRs motifs in nine *Allium* cp genomes.

Supplementary Table S10. The conservation statistic of nine *Allium* cp genome sequences using mVISTA.

Supplementary Table S11. Numbers of nucleotide substitutions and sequence distance in nine complete cp genomes.

Supplementary Table S12. Sites and models in ML and BI analyses for each dataset.

Species	DNA extraction	Sequencing platform	Read length	Assembly software
A. fistulosum	HSLp	Hiseq4000	PE150	NOVOPlasty2.6.2
A. tuberosum Rottl. ex Spreng.	SucDNase	Hiseq4000	PE150	SPAdes 3.11.1
A. sativum	HSLp	Hiseq4000	PE150	NOVOPlasty2.6.2
A. cepa N	PGEK	Hiseq2500	PE100	NOVOPlasty2.6.2

Supplementary Table S1. Basic information about the library, DNA extraction and assembly. HSLp, high-salt low-pH method; SucDNase, sucrose-DNase method; PGEK, Plant Genome Extraction Kit (Tiangen Biotech, Beijing, China).

Sample	Total number of reads	Total number of bases	Mapped to genome (%)	Mean coverage (X)	Number of gaps
A. fistulosum	8,255,274	1,238,291,100	92.36	7,363.85	0
A. tuberosum Rottl. ex Spreng.	13,393,542	2,009,031,300	34.84	4,530.64	2
A. sativum	10,665,090	1,599,763,500	74.05	7,689.11	0
A. cepa N	12,976,420	1,297,642,000	3.99	334.02	0

Supplementary Table S2. Summary of the sequencing data and assembled evaluation for four *Allium* species.

Category for genes	Group of gene	*Name of gene*
Photosynthesis related genes	Photosystem I	*psaA*, *psaB*, *psaC*, *psaI*, *psaJ*
	Photosystem II	*psbA*, *psbB*, *psbC*, *psbD*, *psbE*, *psbF*, *psbH*, *psbI*, *psbJ*, *psbK*, *psbL*, *psbM*, *psbN*, *psbT*, *psbZ*
	Cytochrome b/f complex	*petA*, *petB*, *petD*, *petG*, *petL*, *petN*
	ATP synthase	*atpA*, *atpB*(g/ψ), *atpE*, **atpF*, *atpH*, *atpI*
	Cytochrome c synthesis	*ccsA*
	Assembly/stability of photosystem I	***ycf3*, *ycf4*
	NADPH dehydrogenase	**ndhA*, **ndhB*(2), *ndhC*, *ndhD*, *ndhE*, *ndhF*, *ndhG*, *ndhH*, *ndhI*, *ndhJ*, *ndhK*
	Rubisco	*rbcL*(g/ψ)
Transcription and translation related genes	Transcription	*rpoA*, *rpoB*, **rpoC1*, *rpoC2*
	Translation initiation factor	*ψinfA*(del)
	Ribosomal proteins	*rpl14*, *rpl16*, **rpl2*(2), *rpl20*, *rpl22*, *rpl23*(2), *rpl32*, *rpl33*, *rpl36*, *rps11*, **rps12*(2), *rps14*, *rps15*, **rps16*(g/ψ), *rps18*, *rps19(*2), *rps2*(g/ψ), *rps3*, *rps4*, *rps7*(2), *rps8*
RNA genes	Ribosomal RNA	*rrn16*(2), *rrn23*(2), *rrn4.5*(2), *rrn5*(2)
	Transfer RNA	**trnA-UGC*(2), *trnC-GCA*, *trnD-GUC*, *trnE-UUC*, *trnF-GAA*, *trnfM-CAU*, **trnG-TCC*, *trnG-UCC*, *trnH-GUG*(2), *trnI-CAU*(2), **trnI-GAU*(2), **trnK-UUU*, *trnL-CAA*(2), **trnL-UAA*(g/ψ), *trnL-UAG*, *trnM-CAU*, *trnN-GUU*(2), *trnP-UGG*, *trnQ-UUG*, *trnR-ACG*(2), *trnR-UCU*, *trnS-GCU*, *trnS-GGA*, *trnS-UGA*, *trnT-GGU*, *trnT-UGU*, *trnV-GAC*(2), **trnV-UAC*, *trnW-CCA*, *trnY-GUA*
Other genes	RNA processing	*matK*
	Carbon metabolism	*cemA*
	Fatty acid synthesis	*accD*
	Similarity sequence of *Orf*	*ψorf56*(2), *orf57*(2)
Genes of unknown function	Conserved reading frame	*ψycf15*(2), *ycf1a*, *ycf1b*, *ycf2*(g/ψ)(2), **ycf68*(2)

Supplementary Table S3. List of genes in nine cp genome of *Allium* species. (2), two copies; *, one intron; **,two intron; (g/ψ), gene or pseudogene varied by its genome; (del), absence in *A.tuberosum* Rottl. ex Spreng.

Species	Coding sequences Size (bp)	Coding sequences proportion (%)	rRNA Size (bp)	rRNA proportion (%)	tRNA Size (bp)	tRNA proportion (%)	Pseudogene Size (bp)	Pseudogene proportion (%)
A. fistulosum	80,406	52.50	9,050	5.91	2,859	1.87	1,765	1.15
A. tuberosum Rottl. ex Spreng.	80,391	52.18	9,050	5.87	2,859	1.86	1,550	1.01
A .sativum	80,247	52.38	9,050	5.91	2,859	1.87	2,852	1.86
A. cepa N	80,271	52.26	9,050	5.89	2,859	1.86	1,763	1.15
A. cepa CMS-T	80,421	52.41	9,050	5.90	2,859	1.86	1,759	1.15
A. cepa CMS-S	80,283	52.28	9,050	5.89	2,859	1.86	1,763	1.15
A. obliquum	80,181	52.62	9,050	5.94	2,859	1.88	2,471	1.62
A. prattii	64,419	41.70	9,035	5.85	2,774	1.80	17,782	11.51
A. victorialis	81,072	52.62	9,050	5.87	2,859	1.86	1,043	0.68

Supplementary Table S4. Main components and their proportions in nine *Allium* cp genomes.

Gene content	*A. fistulosum*	*A.tuberosum* Rottl. ex Spreng.	*A. sativum*	*A.cepa* N	*A.cepa* CMS-T	*A.cepa* CMS-S	*A. obliquum*	*A. prattii*	*A. victorialis*
atpB	/	/	/	/	/	/	/	ψ	/
ψinfA	/	del	/	/	/	/	/	/	/
rps16	/	/	ψ	/	/	/	ψ	/	/
rps2	ψ	ψ	ψ	ψ	ψ	ψ	ψ	/	/
rbcL	/	/	/	/	/	/	/	ψ	/
trnL-UAA	/	/	/	/	/	/	/	ψ	/
ycf2(2)	/	/	/	/	/	/	/	ψ	/

Supplementary Table S5. List of the difference in genes content from nine *Allium* cp genome. ψ, pseudogene; /, non-pseudogene; del, absence in *A. tuberosum* Rottl. ex Spreng.; (2), two copies.

Species	Genome	LSC	IR	SSC	Coding sequence	tRNA	rRNA	Pseudogene
A. fistulosum	36.8	34.6	42.7	29.7	37.46	53.06	55.25	39.26
A. tuberosum Rottl. ex Spreng.	36.9	34.7	42.7	29.7	37.59	53.17	55.29	40.00
A. sativum	36.7	34.5	42.6	29.1	37.38	53.10	55.25	35.90
A. cepa N	36.8	34.6	42.7	29.7	37.48	52.99	55.25	39.02
A. cepa CMS-T	36.8	34.6	42.7	29.7	37.46	53.06	55.25	39.28
A. cepa CMS-S	36.8	34.6	42.7	29.7	37.48	52.99	55.25	39.02
A. obliquum	36.8	34.7	42.6	29.4	37.42	52.99	55.23	37.11
A. prattii	37.0	35.0	42.7	29.9	37.36	53.35	55.30	38.92
A. victorialis	37.0	34.9	42.7	30.0	37.70	53.24	55.29	41.04

Supplementary Table S6. GC content (%) of sequence in nine cp genomes of *Allium* species.

	A. fistulosum		*A. tuberosum* Rottl. ex Spreng.		*A. sativum*		*A. cepa* N		*A. cepa* CMS-T		*A. cepa* CMS-S		*A. obliquum*		*A. prattii*		*A. victorialis*	
	GC	length	GC	length	GC	length	GC	length	GC	length	GC	length	GC	length	GC	length	GC	length
ψatpB	/	/	/	/	/	/	/	/	/	/	/	/	/	/	42.34	1502	/	/
ψinfA	35.29	238	del	del	36.70	218	34.05	232	34.05	232	34.48	232	34.05	232	37.56	221	37.1	221
ψorf56(2)	49.43	176	48.86	176	49.43	176	49.43	176	49.43	176	49.43	176	48.86	176	49.43	176	49.43	176
ψrps16	/	/	/	/	30.04	1,102	/	/	/	/	/	/	31.89	715	/	/	/	/
ψrps2	37.31	705	37.64	728	37.61	710	37.09	709	37.09	709	37.59	705	38.18	702	/	/	/	/
ψrbcL	/	/	/	/	/	/	/	/	/	/	/	/	/	/	42.97	1,473	/	/
ψtrnL-UAA	/	/	/	/	/	/	/	/	/	/	/	/	/	/	46.00	50	/	/
ψycf15(2)	36.60	235	37.02	235	36.60	235	36.60	235	36.60	235	36.60	235	36.17	235	36.60	235	36.60	235
ψycf2(2)	/	/	/	/	/	/	/	/	/	/	/	/	/	/	37.92	6,857	/	/
Total	39.26	1,765	40.00	1,550	35.90	2,852	39.02	1,763	39.02	1,763	39.28	1,759	37.11	2,471	38.92	17,782	41.04	1,043

Supplementary Table S7. The content, length (bp) and GC (%) of pseudogene in *Allium* species. (2), two copies; /, non-pseudogene; del, sequence deletion in cp genome.

Species	Tandem repeat	Dispersed repeat	Palindromic repeat	Total number
A. cepa N	19	17	11	47
A. cepa CMS-S	12	13	12	37
A. cepa CMS-T	19	17	11	47
A. fistulosum	15	14	9	38
A. obliquum	21	20	14	55
A. prattii	11	25	13	49
A. sativum	14	18	13	45
A. tuberosum Rottl. ex Spreng.	11	17	11	39
A. victorialis	9	13	15	37
Total number	131	154	109	394

Supplementary Table S8. The numbers of tandem repeats, dispersed, and palindromic in nine *Allium* cp genomes.

Species	Mononucleotide		Dinucleotide		Trinucleotide		Tetranucleotide		Pentanucleotide		Hexanucleotide		Total
	No.	%	No.	%	No.	%	No.	%	No.	%	No.	%	No.
A. fistulosum	62	68.13	13	14.29	3	3.30	10	10.99	3	3.30	0	0.00	91
A. tuberosum Rottl. ex Spreng.	45	61.64	13	17.81	2	2.74	11	15.07	1	1.37	1	1.37	73
A. sativum	53	61.63	18	20.93	1	1.16	12	13.95	2	2.33	0	0.00	86
A. cepa N	65	67.71	15	15.63	3	3.13	10	10.42	3	3.13	0	0.00	96
A. cepa CMS-T	65	67.71	15	15.63	3	3.13	10	10.42	3	3.13	0	0.00	96
A. cepa CMS-S	63	66.32	14	14.74	3	3.16	11	11.58	3	3.16	1	1.05	95
A. obliquum	60	68.97	16	18.39	2	2.30	8	9.20	1	1.15	0	0.00	87
A. prattii	65	68.42	16	16.84	3	3.16	11	11.58	0	0.00	0	0.00	95
A. victorialis	65	68.42	16	16.84	2	2.11	12	12.63	0	0.00	0	0.00	95
Total	543	66.71	136	16.71	22	2.70	95	11.67	16	1.97	2	0.25	814

Supplementary Table S9. Types and numbers of SSRs motifs in nine *Allium* cp genomes.

	Reference length	Reference gene length	Reference Non-gene length	Total conserved sequence	Conserved gene sequence	Conserved Non-gene Sequence
A. fistulosum	154,074	94,024	60,050	97.46%(150,153)	99.43%(93,492)	94.36%(56,661)
A. tuberosum Rottl. ex Spreng.	154,074	94,024	60,050	98.22%(151,339)	99.25%(93,317)	96.62%(58,022)
A. sativum	154,074	94,024	60,050	97.34%(149,976)	99.40%(93,457)	94.12%(56,519)
A. cepa N	154,074	94,024	60,050	97.79%(150,672)	99.46%(93,513)	95.19%(57,159)
A. cepa CMS-T	154,074	94,024	60,050	97.79%(150,672)	99.46%(93,513)	95.19%(57,159)
A. cepa CMS-S	154,074	94,024	60,050	97.90%(150,841)	99.46%(93,513)	95.47%(57,328)
A. obliquum	154,074	94,024	60,050	97.14%(149,667)	99.23%(93,304)	93.88%(56,373)
A. prattii	154,074	94,024	60,050	97.62%(150,401)	99.25%(93,319)	95.06%(57,082)

Supplementary Table S10. The conservation statistic of nine *Allium* cp genome sequences using mVISTA. *A. victorialis* as a reference. The "gene" in column 3 and 6 contain all gene components including the coding gene, tRNA, rRNA and pseudogene. The "Non-gene" in column 4 and 7 are the other.

	A. fistulosum	*A. tuberosum* Rottl. ex Spreng.	*A. sativum*	*A. cepa* N	*A. cepa* CMS-T	*A. cepa* CMS-S	*A. obliquum*	*A. prattii*	*A. victorialis*
A. fistulosum		1,872	1,832	469	464	436	1,178	2,578	2,496
A. tuberosum Rottl. ex Spreng.	0.01265		2,696	1,927	1,922	1,881	2,147	2,060	1,946
A. sativum	0.01238	0.01822		1,879	1,885	1,851	2,123	**3,411**	3,312
A. cepa N	0.00317	0.01303	0.01270		**9**	316	1,222	2,634	2,536
A. cepa CMS-T	0.00314	0.01299	0.01274	**0.00006**		323	1,229	2,627	2,531
A. cepa CMS-S	0.00295	0.01271	0.01251	0.00214	0.00218		1,180	2,583	2,490
A. obliquum	0.00796	0.01451	0.01435	0.00826	0.00831	0.00798		2,838	2,757
A. prattii	0.01743	0.01392	**0.02306**	0.01780	0.01776	0.01746	0.01918		616
A. victorialis	0.01687	0.01315	0.02239	0.01714	0.01711	0.01683	0.01864	0.00416	

Supplementary Table S11. Numbers of nucleotide substitutions and sequence distance in nine complete cp genomes. The upper triangle indicates the number of nucleotide substitutions and the lower triangle indicates the number of sequence distances.

Dataset	Number of sites	Best fit model (AIC)	Model in ML	Model in BI
Complete chloroplast genome	137,185	TPM1uf+G	GTR+G	TPM1uf+G
IRB	24,474	TIM1+I	GTR+G	TPM1uf+I
LSC	71,685	GTR+I+G	GTR+G	TIM1+I+G
SSC	16,157	TVM+G	GTR+G	TPM1uf+G
SC	87,855	GTR+I+G	GTR+G	TPM+I+G
Divergence hot regions	3,724	TIM1+I+G	GTR+G	TPM1uf+I+G

Supplementary Table S12. Sites and models in ML and BI analyses for each dataset.

Supplementary Figures

Supplementary Figure S1. (A) The order of five nodes by alignment with NC_024813 using Mummer 3.23. (B) Location outline of five nodes, two gaps and four PCR primers. Four thin lines from the center represent the boundary of chloroplast quadripartite structures. (C) Agarose gel electrophoresis of the two gaps by PCR amplifications.

Supplementary Figure S2. Number, percentage, and distribution of three types of repeat sites in the cp genomic quadripartite structure (A) and in gene structure (B).

Supplementary Figure S3. Detailed number and distribution of three types of repeats in the IGS, CDS and intron regions of the nine *Allium* cp genomes.

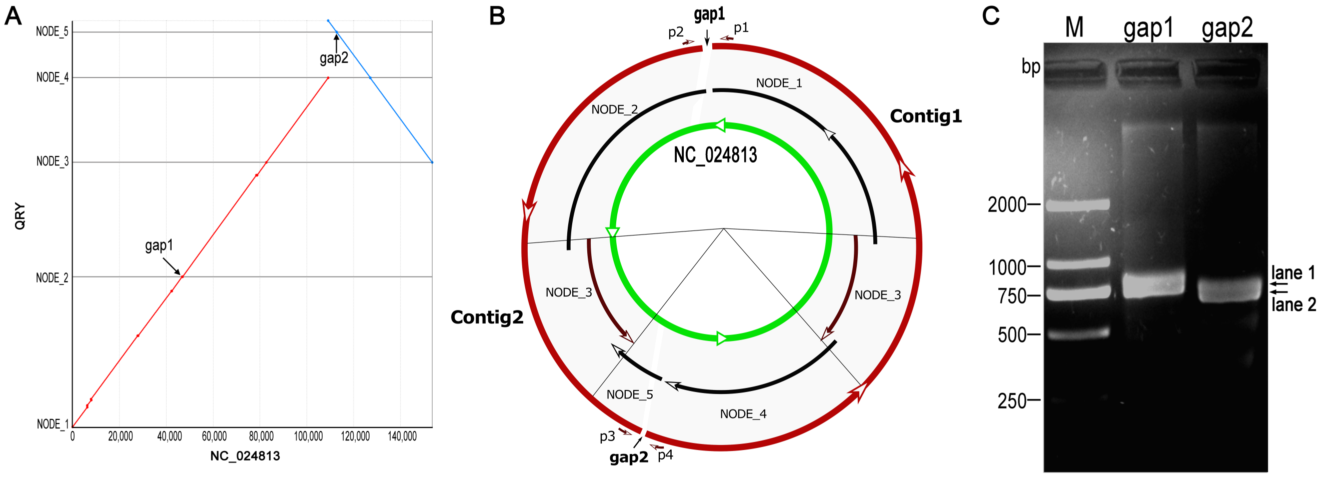

Supplementary Figure S1. (A) The order of five nodes by alignment with NC_024813 using Mummer 3.23. (B) Location outline of five nodes, two gaps and four PCR primers. Four thin lines from the center represent the boundary of chloroplast quadripartite structures. (C) Agarose gel electrophoresis of the two gaps by PCR amplifications.

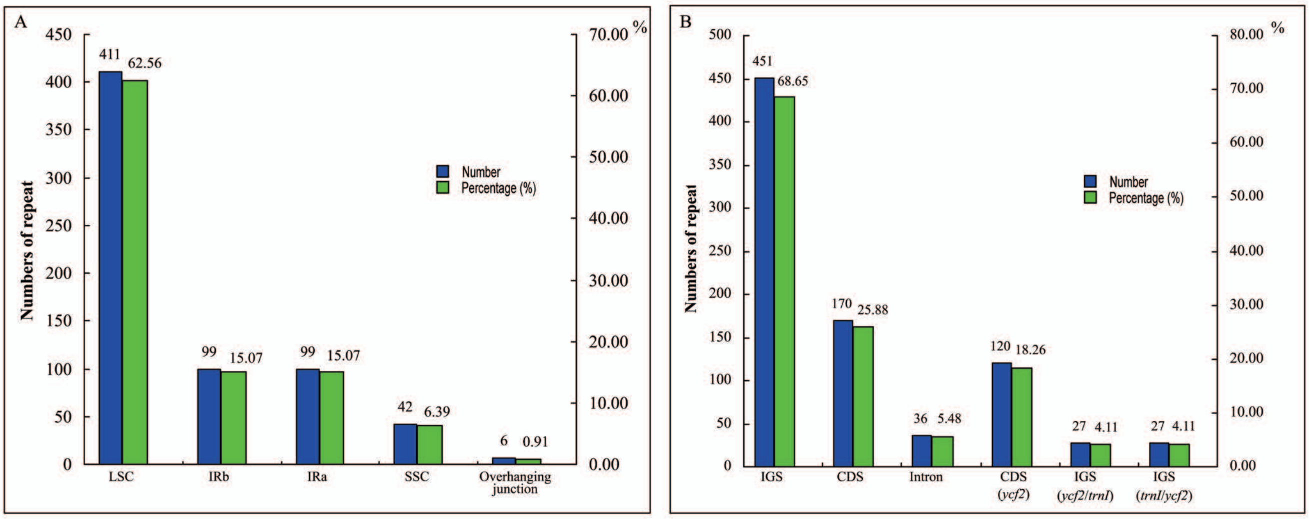

Supplementary Figure S2. Number, percentage, and distribution of three types of repeat sites in the cp genomic quadripartite structure (A) and in gene structure (B).

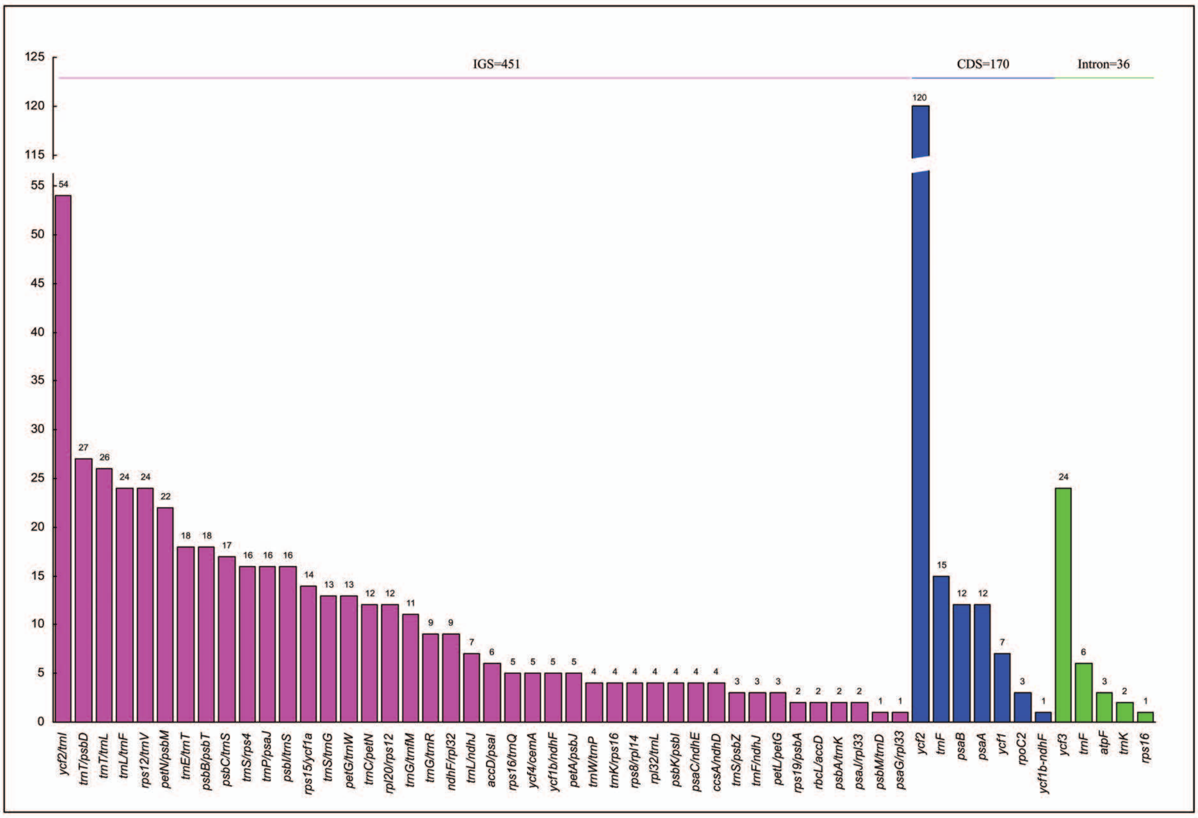

Supplementary Figure S3. Detailed number and distribution of three types of repeats in the IGS, CDS and intron regions of the nine *Allium* cp genomes.

Supplementary datasets

Supplementary Dataset 1 is provided in extra xls file of Supplementary Dataset 1.xls.

Supplementary Dataset 2 is provided in extra xls file of Supplementary Dataset 2.xls.
